# Notes on the Treatment of Skin Diseases

**Published:** 1879-01

**Authors:** 


Notes on the Treatment of Skin Diseases. By Robt. Liveing, A.M. and M.D. Cautab F.R.C.P. London, lately Physician and Lecturer to Middlesex Hospital, etc. Fourth edition revised and enlarged. New York: Wm. Wood & Co., Publishers, 27 Great Jones street; 1878.

This little work of 123 pages will be read with interest by many who find great perplexity in the diagonis and treatment of the various forms of skin diseases. While we have an abiding dislike for formulæ, and while this dogmatical association of remedies decorates the last twenty-three pages of this little book, we nevertheless like the body of the work, which treats of the symptoms, nature and treatment of a troublesome class of diseases.

